# Nortriptyline-Induced Room Tilt Illusion

**DOI:** 10.7759/cureus.52101

**Published:** 2024-01-11

**Authors:** Bernard A Sarmiento, Rojan Varghese, Richa Vijayvargia, Matthew Gunther, Adam Fusick, Shixie Jiang

**Affiliations:** 1 College of Medicine, University of Central Florida, Orlando, USA; 2 Department of Psychiatry and Behavioral Neurosciences, Tampa General Hospital, University of South Florida, Tampa, USA; 3 Department of Psychiatry, University of Florida College of Medicine, Gainesville, USA; 4 Department of Psychiatry, Stanford University School of Medicine, Palo Alto, USA; 5 Mental Health and Behavioral Services, James A Haley VA (Veteran Affairs), Tampa, USA

**Keywords:** visual tilt, verticality, major depressive disorder, major depressive disorder with psychotic features, tricyclic anti-depressants, adverse drug effect, nortriptyline, room tilt illusion

## Abstract

Room tilt illusion (RTI) is a rare and transient perceptual disturbance in which an individual perceives their surroundings as having been rotated or tilted, usually at 90 or 180 degrees. Primarily linked with vestibular disorders and neurological lesions, this report details the only reported occurrence of the RTI phenomena in nortriptyline use for treatment-refractory depression. The patient developed RTI six days after starting the medication and the disturbance resolved after medication cessation. Although the mechanism behind such a phenomenon with medication use has not been elucidated, its etiology may rest on the effect of tricyclic antidepressants on the vestibulo-thalamo-cortical system and visual-vestibular integration. Clinicians should be aware of the potential for such a medication-induced perceptual disturbance, especially in the workup for more serious etiologies in elderly patients with co-morbidities.

## Introduction

Room tilt illusion (RTI), or visual tilt, is a rare and transient disorder of environmental perception resulting in an unusual and disorienting perspective where a patient’s visual scene is rotated off the true vertical [1]. RTI was first described in 1805 when a 22-year-old woman treated for “hysteria” reported hourly episodes where she perceived the people around her as standing on their heads [2]. RTI can vary in duration, but usually episodes last for seconds to hours, occurring mostly in the frontal plane [3].

While the pathophysiological mechanisms underlying RTI remain elusive, it is theorized to be the result of disturbances in verticality perception. Specifically, there is disruption in visual-vestibular integration: erroneous cortical integration of vestibular and visual cues resulting in processing dysfunction [4]. RTI is primarily attributed to conditions affecting the vestibular system and/or vestibular cortical areas, including posterior circulation strokes, labyrinthine disorders, vestibular migraines, and multiple sclerosis [1]. Less commonly, RTI has been associated with non-vestibular conditions such as epilepsy or migraines [5,6]. The 2012 review by Sierra-Hidalgo et al. analyzed 148 cases of RTI noting a male predominance (60.2%), a mean age of 51.2 ± 20.3 years, and etiologies categorized into central nervous system (CNS), vestibular, and peripheral nervous system (PNS) conditions [1]. The study identified posterior circulation cerebral vascular accidents (CVAs) as the most frequent cause of RTI [1].

While much of the existing literature on RTI examines its ties to neurological disorders, its potential association with pharmacological agents remains unexplored. We present a case of nortriptyline-induced RTI in the setting of treatment-refractory depression. Tricyclic antidepressants (TCAs) have been known to induce side effects, such as dizziness, sedation, dry mouth, urinary retention, and blurry vision, from their anticholinergic, histamine-blocking, and α1-adrenergic-blocking properties [7]. Importantly, anticholinergic medications, in general, are associated with visual hallucinations and perceptual alterations [8,9]. Other medications associated with visual perceptual abnormalities include digoxin, PDE_5_ inhibitors, glucocorticoids, benzodiazepines, dopaminergic agents, amphetamines, and opioid agonists [8,9].

Nortriptyline, a TCA known for its overall tolerability, is commonly prescribed for depression, neuropathic pain, and nocturnal enuresis. The occurrence of RTI during therapeutic use with a TCA has yet to be reported. In fact, the current literature has only shown one case of medication-associated RTI after a patient was started on intravenous morphine, suspected to be related to opioid toxicity [1]. Given the scarce documentation and understanding of this phenomenon in relation to medication use, this case report aims to highlight a potential side effect of nortriptyline, discuss the potential effects of TCAs on visual-vestibular integration, and emphasize the importance of a careful diagnostic workup when managing RTI.

## Case presentation

A 77-year-old male patient with a several-month history of worsening depression with psychotic features was transferred to a tertiary academic center for electroconvulsive therapy (ECT). The onset of the patient’s psychiatric symptoms began two years prior after being diagnosed with generalized anxiety disorder (GAD) following the death of his wife. Initial treatments with anxiolytics, as well as supportive counseling, failed to alleviate his symptoms. Instead, he exhibited escalating depression and anxiety, including anorexia with 40-pound weight loss, diminished motivation, hopelessness, anhedonia, and active thoughts of suicide (active suicidal ideation). Psychotic manifestations also emerged later during his depression, including auditory hallucinations of the devil denigrating him and his family and mood-congruent delusional ideas of reference where the patient believed that a TV show was portraying himself as molesting women. Before the emergence of these symptoms, the patient had no past psychiatric history. Medically, he was being treated at that time for atrial flutter (coumadin 5 mg daily), hypertension (losartan 25 mg daily and metoprolol 25 mg twice daily), and obstructive sleep apnea.

Upon hospital admission, his medications included venlafaxine (150 mg daily), aripiprazole (10 mg daily), mirtazapine (30 mg nightly), and trazodone (25 mg as needed). A neurocognitive evaluation was conducted, which ruled out cognitive impairment from delirium or dementia, with a mini-mental state examination yielding a score of 26 of 30 (normal cut-off score of 25). Also, no medical etiologies were identified for his symptoms after a complete physical exam and laboratory testing, including complete blood count, comprehensive metabolic panel, vitamin D, thyroid functioning, and urinalysis. Following eight sessions of ECT, which initially showed promise but regressed after the fourth session, the patient was started on nortriptyline. This was to manage the patient’s severe dysphoria, religious delusions of guilt and financial ruin, and active suicidal ideation without a plan. The venlafaxine and mirtazapine were discontinued, and he began with a 25 mg dose of nortriptyline daily, which was gradually titrated to 50 mg daily over five days. On the sixth day of nortriptyline use, the patient reported very little change to his overall mood, with continued suicidal thoughts and moderate xerostomia. In addition, the patient described a peculiar visual disturbance that morning: it felt as though he was standing on the wall looking downwards as if his surroundings were tilted by 90 degrees. These episodes were intermittent, with no accompanying symptoms, lasting between 2 and 30 seconds, and were solely present for the four weeks he was on nortriptyline. These perceptual abnormalities were not of a hypnopompic nature, as this perceptual abnormality occurred after the patient was fully awake.

The patient’s vital signs were stable over these four weeks and neurological examinations were unremarkable. Computed tomography (CT) and magnetic resonance imaging (MRI) of his head were also unremarkable. After 12 days on nortriptyline, due to profound dysphoria and for post-ECT prophylaxis of treatment-refractory depression, an adjunct treatment with lithium carbonate was initiated, titrated to 600 mg nightly, and the dose of aripiprazole was titrated to 15 mg daily. The lack of mood improvement with augmentation led to the discontinuation of nortriptyline after four weeks. For the two months that he was followed in the hospital upon nortriptyline discontinuation, the patient did not have a recurrence of RTI. The patient was eventually discharged four months after admission, and two months after the nortriptyline trial, with improved depressive symptoms, devoid of suicidal ideation, and minimal mood-congruent delusions of guilt. The patient was discharged on phenelzine 15 mg twice daily, trazodone 25 mg as needed, and was scheduled to be seen by the geriatric psychiatry outpatient clinic within a few weeks.

## Discussion

This is the first described case of RTI occurring in association with tricyclic antidepressant medication use. In terms of medication-induced RTI in general, there has been one other case of medication-induced RTI in a 60-year-old man with adenocarcinoma and multiple bone metastases who developed RTI in close relationship to intravenous morphine initiation and opioid toxicity [1,10].

Understanding the pathophysiology of RTI is important, as neurological etiologies should be considered during workup, even in patients with high suspicion of medication-associated adverse effects. RTI involves dysfunction in how verticality is perceived, which is based on visual-vestibular integration [1]. Verticality, an essential aspect of spatial orientation and balance, refers to the perception of our orientation relative to gravity [11]. Visual-vestibular integration refers to the process through which vestibular, visual, and somatosensory sensory inputs are processed in the CNS to provide a unified perception of spatial orientation [12]. Described as the vestibulo-thalamo-cortical system, sensory information converge for processing in the vestibular nuclei and associated areas, before being projected to the cortex via the ventral lateral and ventral posterior nuclei of the thalamus [12,13]. The cerebellum is also involved in providing spatial orientation information [13,14]. Integration is crucial, as the brain utilizes separate sensory streams to determine the body’s position relative to the environment in order to produce a coherent sense of spatial positioning and orientation and to make appropriate motor adjustments [11-13]. Inner ear conditions, such as benign paroxysmal positional vertigo (BPPV), labyrinthitis, and vestibular neuritis, can affect vestibular sensory input while conditions such as CNS injury can cause integrative processing abnormalities [1,12-13]. RTI in essence is thought to be due to a failure in processing the true vertical at a cortical level due to either a mismatch of sensory inputs or integration disruption [1]. Thus, injuries to any element of this visual-vestibular integration complex can be associated with imbalance, spatial disorientation, and RTI, making anatomical localization challenging, especially with inconclusive neuroimaging. As most cases of RTI are believed to be caused by integrative disruption due to posterior fossa lesions, neurological work-up, including MRI, is critical to work up potential CNS injury, such as infarction and intracranial hemorrhage [1].

Once neurological etiologies have been ruled out, as in the above case, less likely causes should be considered. The theoretical impact of tricyclic antidepressants on the vestibulo-thalamo-cortical system should be discussed, as this could be a potential explanation for the verticality processing dysfunction found in this case of RTI. The mechanisms for TCA's potential vestibular effects are under investigation, are multifaceted, and are presented in Table 1. This table highlights the anticholinergic, histaminergic, and potassium channel-modulating properties of TCAs that can potentially affect the vestibular system.

**Table 1:** Tricyclic antidepressants and their potential effects on the vestibular system Source: [15-18]

Effect	Potential Mechanism of Action
Anticholinergic	Blockade of acetylcholine at muscarinic receptors of the vestibular system can lead to dysfunction, presenting as dizziness and imbalance [15,16]
Histaminergic Modulation	Histaminergic neurotransmission may play a critical role in vestibular function (especially vestibular compensation), and TCA's affinity for antagonizing H_1_ and H_2_ receptors can potentially lead to vestibular dysfunction, although not reported in clinical practice [17]
Potassium Channel Modulation	Studies hint that TCAs can modulate potassium channels; these receptors are thought to play a role in the function of vestibular hair cells [18]

TCAs have also been found to inhibit the reuptake of both norepinephrine and serotonin, increasing their concentration in the synaptic cleft, and thereby modulating a myriad of responses. The role of norepinephrine and serotonin within the vestibular system continues to be explored but they act as chemical mediators within both vestibular and thalamocortical pathways [19]. Originating from the locus coeruleus and nucleus subcoeruleus, noradrenergic fibers have been found to terminate in the vestibular nuclei [19]. Additionally, it has been found that norepinephrine can elicit an excitatory response in a concentration-dependent manner on inferior vestibular nucleus neurons [20]. The inferior vestibular nucleus, as the largest nucleus of the vestibular nuclear complex within the brainstem, receives input from the vestibular nerve, carrying information from the saccule and posterior semicircular canal [21]. The inferior vestibular nucleus then integrates this vestibular information with input from the cerebellum to aid in balance, eye movement, and postural control, functions central for verticality integration. Of note, studies in rats have revealed that norepinephrine can also directly alter neuronal activity in the vestibular complex itself through α2-adrenergic receptor modulation [22,23]. Norepinephrine has been implicated in various aspects of vestibular physiology, such as vestibular compensation and plasticity, stress-related vestibular disorders, and the regulation of blood flow to inner ear structures [24-27].

In terms of serotonergic modulation, vestibular nuclei neurons receive serotonergic projections from the raphe nuclei, leading to a potential modulation in their responsiveness [19,28]. Serotonin receptor 1D (5-HT1DR) and the presynaptic receptor histamine H3 receptor (inhibits both norepinephrine and serotonin release) are expressed in the saccule and semicircular canals of the inner ear [29]. The altered serotonergic function might contribute to conditions such as Méniere’s disease and vestibular migraines, as selective serotonin reuptake inhibitors (SSRIs) and triptans, which elevate serotonin levels in the synaptic cleft, are common therapeutic agents for these conditions [30]. Additionally, serotonin’s role in visual orientation processing within the visual cortex should be highlighted as individuals who use ecstasy/MDMA (3, 4-methylenedioxy-N-methamphetamine), which is known to increase synaptic cleft concentrations of serotonin, are known to experience a tilt aftereffect (TAE) disturbance, suggesting disruptions in orientation and motion processing [31-33]. The TAE phenomenon is where patients experience a transient shift in the perceived orientation of a straight line after being exposed to a tilted line or grating for an extended period of time. Such visual perceptual abnormalities may have pathophysiological overlap with the RTI phenomenon.

Due to TCAs’ broad neuromodulatory effects on the vestibulo-thalamo-cortical system, it is possible that nortriptyline led to the RTI in this patient. Although it is difficult to elucidate causality from one case, the patient had never experienced RTI prior to starting nortriptyline, experienced RTI only after the medication was titrated to 50 mg, and never reported RTI after its discontinuation. Of note, this patient did not experience visual hallucinations or other visual abnormalities during his hospitalization. This is important to point out as patients with schizophrenia spectrum disorders can have complex perceptual disturbances, with tilt aftereffect illusions being described in schizophrenia [34].

The transient and intermittent nature of RTI in this patient is in line with other case reports, which is explained by rapid cortical correction of an unusual perception [35]. Additionally, the patient experienced RTI after fully awakening, which may indicate that a new visual stream could have led to an imbalance in verticality processing that required correction. Finally, most patients with RTI have associated vestibular symptoms, and the patient did report dizziness (with normal blood pressure) upon arising from bed in the morning prior to experiencing the RTI [1,36].

As mentioned, the other case of medication-associated RTI was when a patient was started on intravenous morphine for pain management [1]. Although the exact mechanisms of opioid toxicity on the vestibular system are under investigation, morphine, and synthetic opioids are thought to enhance vestibular sensitivity by activating mu-opioid receptors on the vestibular epithelium [37,38]. Moreover, mu-opioid, delta-opioid, and kappa-opioid receptors have been found in the inner ears of rats [39]. This opioid-associated RTI case further highlights the potential for medication-induced RTI.

This case expands the spectrum of pathologies associated with RTI, as it is one of the only case reports of medication-induced RTI in the literature. Further research is needed to explore both the potential effects of TCAs on vestibulo-thalamo-cortical function and the role of medications in eliciting RTI phenomena.

## Conclusions

RTI is a complex diagnostic challenge for clinicians due to its etiological variety and its rare, transient nature. A disorder of verticality perception, RTI offers an intriguing window into the complex processes underlying spatial processing within the human brain. Understanding these mechanisms can provide valuable insight into the interplay between sensory input and cortical integration, aiding in work-up and management. Although the most common causes of RTI are central or peripheral nervous system lesions, this case report reveals a rare case of medication-induced RTI while highlighting the potential effects of TCAs on the vestibulo-thalamo-cortical system.
